# Stellenwert von Mycophenolat-Mofetil zur Behandlung der interstitiellen Lungenerkrankung bei systemischer Sklerose

**DOI:** 10.1007/s00393-021-01088-y

**Published:** 2021-09-20

**Authors:** Udo Schneider, Elise Siegert, Sven Gläser, Klaus Krüger, die Kommission Pharmakotherapie der DGRh, Andreas Krause

**Affiliations:** 1 Medizinische Klinik mit Schwerpunkt Rheumatologie und Klinische Immunologie, Charité – Universitätsmedizin Berlin, Charitéplatz 1, 10117 Berlin, Deutschland; 2 Berlin Institute of Health, Berlin, Deutschland; 3 Klinik für Innere Medizin – Pneumologie und Infektiologie, Vivantes Klinikum Neukölln, Berlin, Deutschland; 4 Klinik für Innere Medizin – Pneumologie, Vivantes Klinikum Spandau, Berlin, Deutschland; 5 Rheumatologisches Praxiszentrum, München, Deutschland; 6 Klinik für Innere Medizin, Abteilung Rheumatologie, Klinische Immunologie und Osteologie, Immanuel Krankenhaus Berlin, Berlin, Deutschland

**Keywords:** Immunsuppression, Cyclophosphamid, Tocilizumab, Nintedanib, SSc-ILD, Immunosuppression, Cyclophosphamide, Tocilizumab, Nintedanib, SSc-ILD

## Abstract

Die interstitielle Lungenbeteiligung bei systemischer Sklerose (SSc-ILD) ist eine häufige Organkomplikation mit erheblicher Mortalität. Therapeutisch kommen in erster Linie Immunsuppressiva zum Einsatz, insbesondere Cyclophosphamid (CYC) und Mycophenolat-Mofetil (MMF). Neuere Daten zeigen zudem eine Wirksamkeit der Biologika Rituximab und Tocilizumab. Die therapeutischen Optionen wurden zuletzt durch die Zulassung des Antifibrotikums Nintedanib erweitert, dessen Stellenwert insbesondere bei den trotz Immunsuppression progredient fibrosierenden Verläufen der ILD liegt. Die in dieser Übersicht zusammengestellten Daten aus kontrollierten Studien zur Wirksamkeit und Sicherheit von CYC und MMF sprechen für einen bevorzugten Einsatz von MMF. Dem entgegen steht die noch immer fehlende Zulassung von MMF für diese Indikation. Diese wird für eine verbesserte und vereinfachte Versorgung von Patienten mit SSc-ILD dringend benötigt.

## Abstract (en)

Interstitial lung disease in systemic sclerosis (SSc-ILD) is a frequent organ complication with considerable mortality. Therapeutically, immunosuppressants are primarily used, particularly cyclophosphamide (CYC) and mycophenolate mofetil (MMF). Recently acquired data also showed an efficacy of the biologics rituximab and tocilizumab. The therapeutic options have most recently been expanded by the approval of the antifibrotic drug nintedanib. It is particularly beneficial in progressive fibrosing courses of ILD despite immunosuppression. The data from controlled trials on the efficacy and safety of CYC and MMF compiled in this review argue for a preferential use of MMF; however, the approval of MMF for this indication is still lacking. This is urgently needed for improved and simplified care of patients with SSc-ILD.

Interstitielle Lungenerkrankungen (ILD) sind eine heterogene Gruppe von nichtinfektiösen Erkrankungen des Lungenparenchyms und des Interstitiums mit variablen Mustern aus entzündlichen und fibrosierenden Prozessen. Sie verlaufen häufig chronisch progredient und führen dann zu einer fortschreitenden Schädigung und Funktionseinschränkung sämtlicher Kompartimente der Lunge. Viele entzündlich rheumatische Erkrankungen, unter ihnen die systemische Sklerose (SSc), können zu einer Lungenbeteiligung in Form einer ILD führen. Die ILD dominiert dabei nicht selten Morbidität und Mortalität der Patienten.

Die ILD bei systemischer Sklerose (SSc-ILD) ist derzeit die häufigste krankheitsassoziierte Todesursache der Patienten mit SSc [7]. Die medikamentöse Therapie besteht bislang v. a. in der Gabe von Immunsuppressiva, wobei Mycophenolat-Mofetil (MMF) das international am häufigsten verwendete Präparat darstellt [10, 22]. Die in Studien gezeigte Wirksamkeit von MMF auf die SSc-ILD zog nie eine Zulassungsbeantragung für MMF nach sich, sodass es sich in Deutschland formal um eine „Off-label“-Therapie handelt.

Tocilizumab wurde auf Grundlage von Daten einer Phase-III-Studie vor Kurzem von der amerikanischen Zulassungsbehörde FDA zur Behandlung der SSc-ILD zugelassen. Vor diesem Hintergrund soll die vorliegende Übersicht die Evidenz zur Wirksamkeit und Sicherheit sowie den Stellenwert von MMF in der Therapie der SSc-ILD zusammenfassen. Zudem werden die differenzialtherapeutischen Überlegungen zum Einsatz des Antifibrotikums Nintedanib bei SSc-ILD und der insbesondere bei progredientem Verlauf möglicherweise gegenüber einer Monotherapie überlegenen Kombinationstherapie aus Antifibrotikum und Immunsuppressivum diskutiert.

## Hintergrund

Die systemische Sklerose (SSc) ist eine seltene Erkrankung aus der Gruppe der Kollagenosen, die mit einer geschätzten Prävalenz und Inzidenz von 3:10.000 bzw. 0,2:10.000 einhergeht [1] und präferenziell Frauen in mittleren Lebensjahren betrifft.

Die Erkrankung ist klinisch durch ein Mischbild aus oft bereits prodromal vorbestehender Raynaud-Symptomatik, sklerosierenden Hautveränderungen und einem breiten Spektrum an möglichen Organmanifestationen mit u. a. kardiopulmonaler, renaler und gastrointestinaler Beteiligung gekennzeichnet.

Man unterscheidet orientierend eine limitiert kutane (lcSSc) von einer diffus kutanen (dcSSc) Verlaufsform. Während die lcSSc sich klinisch mit distalen Hautmanifestationen präsentiert, typischerweise mit dem Nachweis von Centromer-AK assoziiert ist und häufig zur Entwicklung einer pulmonalarteriellen Hypertonie führen kann, geht die dcSSc mit stammwärts fortschreitender Hautsklerose sowie dem Nachweis von Scl70-AK einher und führt häufiger zu schweren Organmanifestationen, insbesondere einer interstitiellen Lungenerkrankung (SSc-ILD).

Die sklerosierenden Hautveränderungen können klinisch mit dem „modified Rodnan-Skin-Score“ (mRSS) erfasst werden, der als validierter Studienendpunkt eingesetzt wird und dessen Verschlechterung mit einer Progredienz auch extrakutaner Manifestationen und einer erhöhten Mortalität assoziiert ist [41].

## SSc-ILD

Die Prävalenz der interstitiellen Lungenbeteiligung als Komplikation der SSc (SSc-ILD) liegt in einer aktuellen populationsbasierten Kohorte bei etwa 50 % [16]. Vorwiegend sind Patienten mit dcSSC betroffen, die SSc-ILD tritt aber auch bei etwa 30 % der Patienten mit lcSSc auf [12].

Die SSc-ILD beginnt oft mit unspezifischer Allgemeinsymptomatik und dann fortschreitender Belastungsdyspnoe und Husten als führenden Symptomen. In der klinischen Untersuchung ist die Sklerosiphonie, das basal betonte spätinspiratorische Knisterrasseln, ein charakteristischer und häufig nachweisbarer Befund.

Die HRCT der Lunge ist das zentrale Instrument in der Diagnosesicherung und wird aufgrund der Häufigkeit der SSc-ILD mittlerweile auch als Screeninginstrument bei Diagnosestellung einer SSc empfohlen [17]. CT-morphologisch ist eine „nichtspezifische interstitielle Pneumonie“ (NSIP) der häufigste Phänotyp der SSc-ILD mit korrespondierender Histologie, ein UIP(„usual interstitial pneumonia“)-Muster ist seltener [2].

Die neben der CT zur Erfassung einer SSc-ILD und zur Verlaufsbeurteilung wichtigste apparative Diagnostik ist die Lungenfunktionsprüfung einschließlich Blutgasanalyse und Prüfung der Diffusionskapazität für Kohlenmonoxid (DLco). Die prognostisch relevantesten Parameter sind die forcierte Vitalkapazität (FVC) sowie die auf das ventilierte Alveolarvolumen bezogene DLco (Transferkoeffizient, Krogh-Index). Deren relative Abweichungen vom Soll sollten zur Verlaufsbeurteilung regelmäßig seriell untersucht werden, sie dienen zudem als validierte Endpunkte in klinischen Studien. Weiterer prognostisch relevanter Parameter ist die totale Lungenkapazität (TLC). Es ist jedoch wichtig, darauf hinzuweisen, dass es nicht selten falsch negative, also normale Lungenfunktionsbefunde bei bestehender ILD gibt [33]. Als alleiniges Früherkennungsinstrument ist die Lungenfunktionsprüfung somit nicht ausreichend valide.

Der Verlauf der SSc-ILD ist zwar intraindividuell heterogen, aber oft chronisch progredient mit konsekutiver Einschränkung von Lebensqualität und Verkürzung der Lebenserwartung der betroffenen Patienten. Insbesondere Verläufe mit progredienter Fibrosierung stellen daher eine Indikation zur engmaschigen Überwachung und zur medikamentösen Therapie der SSc-ILD dar.

Da bislang keine einheitlichen Standards für die Therapie der SSc-ILD, deren Eskalation bei Progress und das Monitoring der Patienten etabliert sind, werden die Anbindung des Einzelfalls an ein spezialisiertes Zentrum und die dortige Diskussion im interdisziplinären Board empfohlen [17].

## Prognoseabschätzung

Als wegweisend bezüglich der Prognoseabschätzung der SSc-ILD gilt eine nichtinterventionelle Studie [14], in der über einen Zeitraum von 10 Jahren 277 SSc-ILD-Patienten konsekutiv eingeschlossen und verlaufsbeobachtet wurden. Das prozentuale Ausmaß der betroffenen Lungenareale im HRCT, die Reduktion der DLco und der Nachweis einer pulmonalen Hypertonie waren unabhängig voneinander mit einer erhöhten Mortalität assoziiert. Mit der Kombination aus HRCT (mit einem Schwellenwert von 20 % bezüglich des Ausmaßes von durch die ILD betroffenen Lungenarealen) und FVC (mit einem Schwellenwert von 70 % bei grenzwertigem HRCT) konnte ein einfaches Scoring-System entwickelt werden („limited“ vs. „extensive disease“), mit dem eine erhöhte Mortalität besser abgeschätzt werden konnte als mit alleiniger Berücksichtigung des HRCTs oder einzelner Lungenfunktionsparameter [14]. Für Patienten mit dcSSc konnte eine Korrelation zwischen progredienten Hautveränderungen und Verschlechterung von Lungenfunktion und Überleben in einer europäischen Kohorte gezeigt werden [41].

## Therapie

Trotz der prognostisch großen Bedeutung der SSc-ILD für den Verlauf einer SSc liegen bisher nur einzelne kontrollierte Studien zur Behandlung vor. Grundlage der medikamentösen Therapie der SSc-ILD bestand bislang – dem autoimmunen Charakter der Erkrankung entsprechend – v. a. in der Gabe unterschiedlicher Immunsuppressiva. Aktuelle Studien zur Behandlung der SSc mit Tocilizumab und Rituximab geben erstmals Hinweise auf eine Wirksamkeit dieser Biologika auch auf die SSc-ILD. Zudem wurde die Wirkung des Antifibrotikums Nintedanib bei SSc-ILD nachgewiesen, und die therapeutischen Möglichkeiten wurden um ein neues Therapieprinzip erweitert (Tab. 1).

**Table 1:** 

Studienname [Referenz] Teilnehmerzahl *n*	Design	(Wesentliche) Einschlusskriterien	Ergebnis primärer Endpunkt	%FVC als sek. Endpunkt	Evidenzgrad
**SLS I **[36] (*n* = 158)	Randomisierte, doppelblinde, placebokontrollierte Studie	SSc nach ACR-Kriterien 1980	Differenz in der adjustierten absoluten Veränderung der FVC % v. Soll Baseline vs. 12 Monate: 2,53 % (95 % CI, 0,28–4,79 %) zugunsten von CYC (*p* < 0,03)	n. z.	Ib
	**CYC p.o.** (2 mg/kg) oder Placebo für 12 Monate + 12 Monate Follow-up ohne Therapie	Erstes Nicht-Raynaud-Symptom <7 Jahre			
		Nachweis einer aktiven Alveolitis			
		Belastungs-Dyspnoe-Grad ≥2 auf der Magnitude of Task-Komponente des Mahler-Baseline-Dyspnoe-Index			
**SLS II **[37] (*n* = 142)	Randomisierte, doppelblinde, kontrollierte Studie	SSc nach ACR-Kriterien 1980	Kein signifikanter Unterschied zwischen MMF und CYC bezüglich FVC % v. Soll im Monat 24 (Unterschied zwischen den Gruppen: −0,70 [95 % Cl −3,1, 1,7]; *p* = 0,56)	n. z.	Ib
	**CYC p.o.** (2 mg/kg) für 12 Monate + 12 Monate Placebo oder **MMF p.o. **(3 g/Tag) für 24 Monate	Erstes Nicht-Raynaud-Symptom <7 Jahre			
		Milchglastrübung im HRCT mit oder ohne assoziierte Retikulationen			
		Belastungs-Dyspnoe-Grad ≥2 auf der Magnitude of Task-Komponente des Mahler-Baseline-Dyspnoe-Index			
		FVC ≥45 %–≤80 %/Soll			
		DLCO ≥40 %/Soll; ≥30 % und <40 % ohne signifikante PH			
**focuSSced **[21] (*n* = 212)	Randomisierte, doppelblinde, placebokontrollierte Studie	SSc nach ACR/EULAR-Kriterien 2013	Keine Verbesserung des mRSS vs. Baseline in Woche 48	Nichtverschlechterung %FVC gegenüber Baseline 4,2 zugunsten TCZ (95 % CI 2,0–6,4; *p* = 0,0002)	Ib
	Doppelblinde Studienphase: **TCZ **(162 mg sc. QW) oder Placebo für 48 Wochen	Erstes Nicht-Raynaud-Symptom ≤60 Monate	TCZ-Arm zeigte numerisch stärkere Reduktion im mRSS (−1,7; 95 % CI −3,8–0,3; *p* = 0,10)		
	Open-Label: TCZ (162 mg, sc. QW für 48 Wochen)	dcSSc, mRSS 10–35			
		Mindestens eins der folgenden: CRP ≥6 mg/l, ESR ≥28 mm/h, Thrombozyten ≥330 × 10^9^/l			
**SENSCIS **[5] (*n* = 576)	Randomisierte, doppelblinde, placebokontrollierte Studie	SSc nach ACR/EULAR-Kriterien 2013	Rückgang der adjustierten jährlichen FVC-Rate über 52 Wochen: 41,0 ml/Jahr (95 % CI, 2,9–79,0) zugunsten von Nintedanib; *p* = 0,04; relative Reduktion von 44 %	n. z.	Ib
	**Nintedanib** p.o. 150 mg 2‑mal/Tag vs. Placebo für mindestens 52 Wochen	Erstes Nicht-Raynaud-Symptom <7 Jahre			
		ILD im HRCT ≥10 %			
		FVC ≥40 %/Soll			
		DLCO 30–89 %/Soll			
**DESIRES **[6] (*n* = 56)	Randomisierte, doppelblinde, placebokontrollierte Studie	SSc nach ACR/EULAR-Kriterien 2013	Rückgang des mRSS unter RTX −6,3 vs. +2,14 unter PCB (95 % CI, −11 bis −5,88, *p* < 0,0001)	+0,1 % vs. −2,9 % zugunsten RTX, (95 % CI, 0,08–5,84 %, *p* = 0,04)	Ib
	4‑mal 375mg/m^2^ RTX i.v. vs. Placebo	mRSS ≥10			
	Read-out nach 24 Wochen	FVC >60 %/Soll			

*SLS* Scleroderma Lung Study, *CYC* Cyclophophsamid, *p. o.* per os, *MMF* Mycophenolatmofetil, *TCZ* Tocilizumab, *sc.* subkutan, *QW* wöchentlich, *RTX* Rituximab, *i. v.* intravenös, *SSc* systemische Sklerose, *ACR* American College of Rheumatology, *FVC* funktionelle Vitalkapazität, *DLCO* Diffusionskapazität, *PH* pulmonale Hypertonie, *dcSSc* diffus kutane systemische Sklerose, *mRSS* „modified Rodnan Skin Score“, *CRP* C-reaktives Protein, *ESR* Blutsenkungsgeschwindigkeit, *ILD* interstitielle Lungenerkrankung, *HRCT* hochauflösende Computertomographie, *CI* Konfidenzintervall, *PCB* placebo, *n.z.* nicht zutreffend

Bei der Interpretation vorliegender Studiendaten muss neben den sehr heterogenen formalen Qualitätskriterien (z. B. multizentrisch prospektiv doppelblind placebokontrolliert vs. monozentrisch retrospektiv, Patientenzahl, Beobachtungsdauer) auch berücksichtigt werden, dass die jeweiligen patienten- und krankheitsspezifischen Ein- und Ausschlusskriterien (Krankheitsdauer, Antikörperstatus, Entzündungsparameter, Hautscore, (Nicht‑)Ansprechen auf Vortherapien, Art und Ausmaß der CT-morphologischen und lungenfunktionellen Veränderungen zur Baseline) entscheidenden Einfluss auf die Ergebnisse haben.

Die Evidenz für einzelne medikamentöse Therapien und die autologe Stammzelltransplantation wird im Folgenden nur kursorisch und für MMF ausführlich dargelegt. An dieser Stelle sei betont, dass außer Nintedanib keines der hier genannten Medikamente in Deutschland zur Behandlung der SSc-ILD zugelassen ist.

*Glukokortikoide *(GC) spielen in der Therapie der SSc eine untergeordnete Rolle, seit ihre höher dosierte Gabe (>15 mg Prednisolonäquivalent) als Risikofaktor für die Entwicklung der „renalen Krise“, eines akuten SSc-spezifischen Nierenversagens mit hoher Mortalität, identifiziert wurde [32].

Unabhängig hiervon wurde ein Wirksamkeitsnachweis für die SSc-ILD in randomisierten kontrollierten Studien nie erbracht, auch wenn eine offene Studie einen positiven Effekt für höhere GC-Dosen beschreibt [26] und niedrig-dosierte GC begleitend zu konventioneller Immunsuppression in Studien eingesetzt wurden [18].

*Methotrexat* (MTX) wird zwar in der Therapie der frühen dcSSc häufig eingesetzt und diesbezüglich in den EULAR-Empfehlungen erwähnt [23], spielt aber in der Therapie der SSc-ILD aufgrund eines fehlenden Wirksamkeitsnachweises keine Rolle.

*Azathioprin* wird v. a. sequenziell nach Cyclophosphamid eingesetzt, diesbezüglich liegen Daten aus einer kontrollierten Studie vor [18]. Als primäre Therapie war Azathioprin mit signifikanter Verschlechterung der Lungenfunktionsparameter im direkten Vergleich zu Cyclophosphamid in einer offenen Studie unterlegen [24].

Für *Rituximab *(RTX) liegen positive Ergebnisse aus einer offenen kontrollierten Studie im Vergleich zu Cyclophosphamid bei früher SSc-ILD vor [31]. Weitere Daten zur Wirksamkeit bei SSc-ILD stammen aus einer monozentrischen Studie in Kombination mit MMF [13], aus einer retrospektiven französischen Studie für die SSc [37] sowie aus einer EUSTAR-Observationsstudie [7], aufgrund derer die B‑Zell-Depletion zumindest als Reserveoption bei therapierefraktären Verläufen angesehen wird [17]. Ganz aktuell wurde eine placebokontrollierte randomisierte japanische Studie publiziert, in der RTX nach 24 Wochen zu einer Verbesserung des mRSS als primärem Endpunkt, aber auch zu einer Verbesserung der FVC als sekundärem Endpunkt führte [6]. Im korrespondierenden Editorial wird jedoch auf die Notwendigkeit größerer, a. e. internationaler Studien zur Erhärtung der Daten hingewiesen, zumal die Verschlechterung des mRSS in der Placebogruppe anhand vergleichbarer Kollektive so nicht erwartet worden wäre [19]. Bei der Interpretation der Daten bezüglich der FVC muss berücksichtigt werden, dass das Vorliegen einer SSc-ILD kein Einschlusskriterium war.

Ein positiver Einfluss von *Tocilizumab (TCZ)* auf die FVC von SSc-Patienten konnte in 2 randomisierten placebokontrollierten Studien gezeigt werden. Die Studien waren nicht auf die SSc-ILD ausgerichtet, sondern hatten eine Verbesserung des mRSS als primären Endpunkt, der nicht erreicht wurde. Die FVC zeigte in beiden Studien unter Tocilizumab im Verlauf eine Stabilisierungstendenz und nahm bei etwa der Hälfte der Patienten über 48 Wochen nicht ab. Unter Placebo hingegen erlitten über 70 % der SSc-ILD-Patienten eine z. T. deutliche Abnahme der FVC [20, 21]. Die Daten führten im März 2021 zur Zulassung der 1‑malig wöchentlichen subkutanen Gabe von Tocilizumab zur Therapie der SSc-ILD durch die FDA.

Die *autologe Stammzelltransplantation* (ASCT) konnte in mehreren Studien gute Resultate gegenüber „standard of care“ zeigen [3, 39], ist jedoch mit einer nicht unerheblichen Mortalität behaftet, was die rechtzeitige Selektion geeigneter Patienten mit positiv zu bewertender Nutzen-Risiken-Kalkulation bei zusätzlich eingeschränkter Verfügbarkeit erschwert [9]. Die Methode bleibt daher zurzeit der Behandlung schwerer therapierefraktärer Krankheitsverläufe mit schlechter Prognose vorbehalten.

*Cyclophosphamid *(CYC) hat in einer großen kontrollierten randomisierten Studie (Scleroderma Lung Study/SLS I) seine Überlegenheit gegenüber Placebo zeigen können [35]. Die 1‑jährige Gabe von oralem Cyclophosphamid führte zu einer Verbesserung des primären Studienendpunkts FVC von 2,5 %. Dieser therapeutische Vorteil gegenüber Placebo war allerdings nach einem Nachbeobachtungsjahr in der Verumgruppe nicht mehr nachweisbar, was aber möglicherweise auch durch das Fehlen einer vorgegebenen sequenziellen Erhaltungstherapie erklärt werden kann.

In einer weiteren placebokontrollierten Studie mit CYC als intravenöser Bolustherapie für 6 Monate kombiniert mit niedrig dosierten Glukokortikoiden gefolgt von Azathioprin konnte nach 1 Jahr ebenfalls ein positiver Effekt auf die FVC im Sinne eines Zuwachses von 4,1 % gegenüber den Ausgangswerten gezeigt werden, allerdings bei einer vergleichsweise hohen Zahl von Studienabbrüchen von fast 40 % und knapp verfehltem Signifikanzniveau [18].

Aufgrund der Ergebnisse der beiden genannten Studien zählt CYC in Deutschland zu den Standardtherapien bei SSc-ILD. Trotz fehlender expliziter Zulassung für diese Indikation wird diese Therapie wie bei anderen bedrohlich verlaufenden Autoimmunerkrankungen auch von den Kostenträgern anerkannt. Die aktuellen Empfehlungen der European League Against Rheumatism (EULAR) zur Therapie der SSc-ILD beinhalten CYC mit dem Empfehlungsgrad A [23].

## Mycophenolat-Mofetil (MMF)

MMF ist ein selektiver und reversibler Hemmer der Inosinmonophosphat-Dehydrogenase, der die Synthese des für die Proliferation von T‑ und B‑Lymphozyten wichtigen Purins Guanosin hemmt. Da Lymphozyten über einen gering ausgeprägten Wiederverwertungsstoffwechsel von Guanosin verfügen, wirkt MMF auf sie stärker zytostatisch als auf andere Zellen [29].

MMF ist ein Prodrug, das in den aktiven Metaboliten Mycophenolsäure (MPA) umgewandelt wird.

In den meisten Studien wurde MMF eingesetzt. MPA zeichnet sich bei gleicher Wirksamkeit durch eine bessere gastrointestinale Verträglichkeit aus.

Initial zugelassen wurde MMF zur Vermeidung von Abstoßungsreaktionen in der Transplantationsmedizin, hat im Verlauf aber auch in der Rheumatologie bei der Therapie verschiedener refraktärer Autoimmunerkrankungen eine zentrale Rolle eingenommen, insbesondere in der Therapie der proliferativen Lupusnephritis [8]. Letztere Indikation ist die einzige, für die MMF bisher in der Rheumatologie in Deutschland eine Zulassung erhalten hat.

Die stärkste Evidenz zur Wirksamkeit von MMF bei SSc-ILD beruht auf den Daten aus der randomisierten kontrollierten doppelblinden „Scleroderma Lung Disease 2“(SLS II)-Studie. In dieser wurden 2 Jahre einer MMF-Therapie (2-mal 1500 mg) mit 1 Jahr oralem CYC (2,0 mg/kgKG) gefolgt von 1‑jähriger Placebotherapie verglichen [36].

Die wesentlichen Ein- und Ausschlusskriterien umfassten (ähnlich wie in SLS I, s. auch Tab. 1) eine FVC von mindestens 45 % und höchstens 80 %, eine DLco von mindestens 40 %, den Nachweis interstitieller Lungenveränderungen im HRCT, den Ausschluss einer pulmonalen Hypertonie und eine Krankheitshöchstdauer von 7 Jahren (nach erstem Auftreten einer anderen Symptomatik als Raynaud).

Primärer Endpunkt war die Veränderung der FVC nach 24 Monaten, sekundäre Endpunkte waren die Veränderung der DLco, des mRSS und der Transition Dyspnea Index (TDI).

In beiden Therapiearmen (MMF: 69 Patienten, CYC: 73 Patienten) fand sich ein positiver Effekt auf die FVC nach 24 Monaten. Durchschnittlich nahm die FVC intraindividuell gegenüber dem jeweiligen Ausgangsbefund um 2,88 % (95 % CI 1,19–4,58) im CYC- und um 2,19 % (95 % CI 0,53–3,84) im MMF-Arm zu.

**Figure 1:**
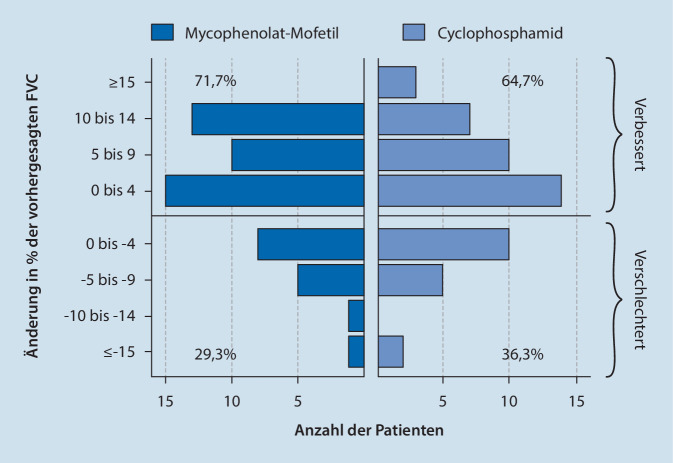

Betrachtet man die Verteilung der Patienten beider Therapiearme, deren Daten nach 24 Monaten verfügbar waren, bezüglich Intervallen von ∆5 %FVC wird ersichtlich, dass einzelne Patienten, die eine Verbesserung von >15 %FVC erreicht haben, die Gesamtauswertung zugunsten des CYC-Armes beeinflusst haben, wohingegen sich gesamtanteilig mehr Patienten unter MMF verbessern bzw. weniger verschlechtern als unter CYC (s. Abb. 1).

Zusätzlich gingen 15 in den CYC-Arm randomisierte Patienten in diese Auswertung ein, die die Studienmedikation beendet hatten und mit anderen Immunsuppressiva (u. a. MMF) weiterbehandelt worden waren. MRSS und TDI besserten sich gleichsinnig in beiden Therapiearmen.

Die durchschnittliche intraindividuelle Abnahme der DLco war unter MMF geringer (−0,4 %) als unter CYC (−2,12 %). Der primäre Endpunkt (Überlegenheit MMF gegenüber CYC bezüglich %FVC nach 24 Monaten) wurde somit nicht erreicht.

Bezüglich Sicherheit waren Leuko- und Thrombopenien im CYC-Arm (41 % bzw. 6 %) häufiger als im MMF-Arm (6 % bzw. 0 %), ebenso therapieassoziierte schwere unerwünschte Wirkungen (SAEs) (10 % CYC, 4 % MMF) und Todesfälle (15 % im CYC-, 7 % im MMF-Arm, diese traten jedoch jeweils ganz überwiegend nach Beendigung der Studienmedikation ein). Die Gesamtzahl der Studienabbrüche war im CYC-Arm höher (36 vs. 20), und sie erfolgten frühzeitiger. Insgesamt zeigte MMF somit in der SLS II bei vergleichbarer Wirksamkeit ein besseres Sicherheitsprofil als CYC.

In einer weiteren Arbeit wurde – mit Hinweis auf die Ähnlichkeit der Ein- und Ausschlusskriterien und die dadurch vergleichbaren Patientenpopulationen – der MMF-Arm aus der SLS II mit dem Placeboarm der SLS I verglichen. Bei erwartbarer Überlegenheit von MMF gegenüber Placebo bezogen auf die %FVC war dieser positive Effekt auch nach 24 Monaten noch vorhanden, was für orales CYC in der ursprünglichen Studie nicht hatte gezeigt werden können [40].

Weitere Daten für die vergleichbare Wirksamkeit von MMF im Vergleich zu Cyclophosphamid bei SSc-ILD liegen aus monozentrischen Studien vor [27, 30], darüber hinaus wurde in Kohortenstudien bei Kollagenosen mit interstitieller Lungenerkrankung (CTD-ILD) über die gute Wirksamkeit und Sicherheit auch bezüglich der jeweils enthaltenen Subgruppe der SSc-ILD berichtet [11, 34].

Im Vergleich von CYC und MMF muss neben der Wirksamkeit auch die Toxizität der einzelnen Substanzen berücksichtigt werden. CYC ist teratogen, mutagen und kann unabhängig hiervon zu einer Ovarialinsuffizienz mit frühzeitiger Menopause und der Gefahr von Spätkomplikationen (u. a. Osteoporose) führen [28]. Die in den SLS I und II angewandte orale CYC-Therapie gilt in der heutigen klinischen Routine aufgrund der CYC-Toxizität als obsolet. Mit einer täglichen Dosis von 2 mg/kgKG und 1‑jähriger Therapie werden gängige Empfehlungen bezüglich der maximalen kumulativen Dosis von CYC erreicht bzw. überschritten [25], ohne damit im Therapiezeitraum das Problem der SSc-ILD nachhaltig gelöst zu haben. Ob die in der Behandlungsrealität alternativ bevorzugte intravenöse CYC-Bolustherapie eine vergleichbare Wirkung wie orales CYC bzw. MMF hat, ist nach den klinischen Erfahrungen zu vermuten, wurde aber nie in einer Studie gezeigt. Die Problematik einer kumulativen Toxizität oder einer daraus resultierenden zeitlichen Limitierung der maximalen Behandlungsdauer besteht bei MMF nicht.

Schließlich bleibt zu berücksichtigen, dass die CYC-Bolustherapie oft stationär durchgeführt wird. Eine als zumindest gleichwertig anzusehende MMF-Therapie erscheint daher auch hinsichtlich ökonomischer Aspekte und Inanspruchnahme von Ressourcen sinnvoller zu sein als eine Therapie mit CYC.

## Bestehende Empfehlungen zur Therapie der SSc-ILD

Es liegen zahlreiche nationale und internationale Empfehlungen zur medikamentösen, immunsuppressiven Behandlung der SSc-ILD vor.

Das Update der EULAR-Empfehlungen von 2017 für die Therapie der SSc empfiehlt, aufgrund der zum Zeitpunkt der Publikation vorliegenden Studienergebnisse der 2 entsprechenden randomisierten kontrollierten Studien [18, 35] die Gabe von CYC bei SSC-ILD in Erwägung zu ziehen [23].

Alternativ wird die ASCT als Option bei refraktären Verläufen erwähnt. Die Ergebnisse der SLS II lagen zum Zeitpunkt der Datensichtung als Grundlage für diese Empfehlungen noch nicht vor. MMF wird in diesen Empfehlungen noch in der Research Agenda erwähnt, weil die entsprechenden Studiendaten ausstanden.

Die britische Gesellschaft für Rheumatologie empfiehlt MMF als Alternative zu CYC bei SSc-ILD [4].

In einem US-amerikanischen interdisziplinären Konsensus bezüglich Management und Therapie der SSC-ILD wurde MMF als Primärtherapie gesehen [22].

Ebenso empfiehlt die „Scleroderma Algorithm Group“ MMF sowohl in der Induktions- als auch in der Erhaltungstherapie der SSC-ILD als Erstlinienpräparat [10].

In Japan wurde die Zulassung für MMF bei SSc-ILD beantragt [38].

Die aktuellsten Konsensusempfehlungen als Ergebnis eines moderierten Delphi-Prozesses stammen von einem interdisziplinären europäischen Expertengremium. Dieses sieht die Gabe von MMF und CYC als gleichwertig als Erstlinientherapie bei behandlungsbedürftiger SSc-ILD an [17]. Rituximab und Stammzelltransplantation folgen als Empfehlungen in therapierefraktären Fällen. Tocilizumab wird aufgrund der erst kürzlich publizierten Daten nicht erwähnt.

## Antifibrotische Therapie

Neben der nachgewiesenermaßen wirksamen immunsuppressiven Behandlung der SSc-ILD besteht die Möglichkeit einer antifibrotischen Therapie der SSc-ILD. Für diesen bei der idiopathischen Lungenfibrose etablierten Therapieansatz steht Nintedanib als zugelassenes Medikament zur Verfügung.

In der randomisierten doppelblinden placebokontrollierten „Scensis“-Studie wurde die antifibrotische Wirksamkeit des Tyrosinkinaseinhibitors Nintedanib bei SSc-ILD bezüglich der geringeren Abnahme der FVC nach 1 Jahr als primärer Endpunkt untersucht.

Nintedanib führte im Vergleich zu Placebo zu einer statistisch signifikant geringeren Abnahme der FVC im Beobachtungszeitraum [5].

Der geringste FVC-Verlust fand sich in der Subgruppe der parallel mit MMF behandelten Patienten [15], auch wenn die Studie nicht auf den Wirksamkeitsnachweis einer solchen Kombinationstherapie ausgerichtet war.

Relevant häufigere oder schwerere Nebenwirkungen wurden unter der Kombinationstherapie von Nintedanib mit MMF im Vergleich zu Nintedanib ohne die immunsuppressive Begleitmedikation nicht beobachtet, obwohl es unter beiden Präparaten in Monotherapie häufig zu gastrointestinalen Nebenwirkungen kommt.

Immunsuppressive und antifibrotische Behandlung stellen also zwar unterschiedliche, sich aber nicht gegenseitig ausschließende, sondern im Einzelfall synergistisch wirksame Therapieansätze dar. Welche Patienten mehr von der einen oder der anderen oder der Kombinationstherapie profitieren, ist derzeit noch nicht klar.

## Fazit und Empfehlung

Die SSc-ILD ist eine mit erheblicher Mortalität behaftete Organmanifestation der SSc. Die immunsuppressive Behandlung der SSc-ILD hat sich als wirksam erwiesen und wird entsprechend in nationalen und internationalen Richtlinien empfohlen. Daneben bzw. ergänzend steht neu das Antifibrotikum Nintedanib als zugelassene Therapieoption der SSc-ILD zur Verfügung.

Unter den Immunsuppressiva sind den vorliegenden Studiendaten entsprechend MMF, CYC und seit Neuestem auch Tocilizumab die Präparate mit der besten Evidenz aus kontrollierten klinischen Studien.

Die Wirksamkeit von MMF und CYC bei SSc-ILD scheinen nach der SLS II vergleichbar zu sein. Direkte oder indirekte Vergleiche zwischen MMF bzw. CYC und Tocilizumab liegen nicht vor.

MMF hat gegenüber CYC den Vorteil des sowohl kurz- als auch langfristig besseren Sicherheitsprofils und es ist einfacher applizier- und steuerbar. Tocilizumab scheint nach bisher vorliegenden Daten bei der SSc-ILD gut verträglich zu sein.

Eine synergistische Wirksamkeit der Kombination aus Antifibrotikum und Immunsuppressivum ist nicht bewiesen, aber plausibel. Vergleichbare Studiendaten zur Kombinationstherapie mit Nintedanib liegen weder für CYC noch für das im März 2021 von der FDA zugelassene Tocilizumab vor.

Aufgrund der derzeit vorliegenden Daten und der breiten klinischen Erfahrung stellt MMF das Immunsuppressivum der ersten Wahl zur Therapie der SSc-ILD dar. CYC bleibt eine Alternative. Die Differenzialindikation zwischen diesen Präparaten muss individuell unter Einschluss weiterer Organmanifestationen, Verträglichkeit und Patientenpräferenz getroffen werden. Tocilizumab, obwohl genau wie MMF zur Behandlung der SSc-ILD in Deutschland nicht zugelassen, stellt eine therapeutische Alternative für Patienten, die auf eine Behandlung mit MMF und CYC nicht ausreichend ansprechen oder diese nicht vertragen, dar. Weitere Studien werden notwendig sein, um den Stellenwert der 3 genannten Substanzen einschätzen und deren optimalen Einsatz festlegen zu können.

Der Einsatz von MMF bei SSc-ILD – obwohl international Standard – wird in Deutschland durch die fehlende Zulassung von MMF in dieser Indikation erschwert. Regional wird die Kostenerstattung durch die Krankenkassen sehr unterschiedlich gehandhabt und reicht von Akzeptanz als Medikament der Wahl und somit Kostenerstattung über Prüfung durch den Medizinischen Dienst im Einzelfall bis zur Ablehnung der Kostenübernahme. Dies betrifft auch Fälle, in denen CYC erfolglos eingesetzt worden ist. Gerade Letzteres ist im Hinblick auf diese ebenfalls nicht explizit zugelassene Behandlung und die vergleichbare Wirksamkeit von MMF und CYC wissenschaftlich nicht nachvollziehbar. Im Falle einer Zulassung von Tocilizumab zur Behandlung der SSc-ILD auch in Europa wird damit der Einsatz von MMF in dieser Indikation weiter deutlich erschwert werden, obwohl diese Therapie nachgewiesenermaßen wirksam, verträglich und im therapeutischen Spektrum inzwischen unentbehrlich ist. Die Verordnungsfähigkeit von MMF in der Indikation SSc-ILD würde daher die Behandlung von Patienten mit SSc-ILD in Deutschland verbessern, vereinfachen und dem internationalen Standard anpassen.

## References

[1] Andréasson K, Saxne T, Bergknut C (2014). Prevalence and incidence of systemic sclerosis in southern Sweden: population-based data with case ascertainment using the 1980 ARA criteria and the proposed ACR-EULAR classification criteria. Ann Rheum Dis.

[2] Bouros D, Wells AU, Nicholson AG (2002). Histopathologic subsets of fibrosing alveolitis in patients with systemic sclerosis and their relationship to outcome. Am J Respir Crit Care Med.

[3] Burt RK, Shah SJ, Dill K (2011). Autologous non-myeloablative haemopoietic stem-cell transplantation compared with pulse cyclophosphamide once per month for systemic sclerosis (ASSIST): an open-label, randomised phase 2 trial. Lancet.

[4] Denton CP, Hughes M, Gak N (2016). BSR and BHPR guideline for the treatment of systemic sclerosis. Rheumatology.

[5] Distler O, Highland KB, Gahlemann M (2019). Nintedanib for systemic sclerosis-associated interstitial lung disease. N Engl J Med.

[6] Ebata S, Yoshizaki A, Oba K (2021). Safety and efficacy of rituximab in systemic sclerosis (DESIRES): a double-blind, investigator-initiated, randomised, placebo-controlled trial. Lancet Rheumatol.

[7] Elhai M, Boubaya M, Distler O (2019). Outcomes of patients with systemic sclerosis treated with rituximab in contemporary practice: a prospective cohort study. Ann Rheum Dis.

[8] Fanouriakis A, Kostopoulou M, Cheema K (2020). 2019 update of the joint European league against rheumatism and European renal association-European dialysis and transplant association (EULAR/ERA-EDTA) recommendations for the management of lupus nephritis. Ann Rheum Dis.

[9] Farge D, Burt RK, Oliveira MC (2017). Cardiopulmonary assessment of patients with systemic sclerosis for hematopoietic stem cell transplantation: recommendations from the European society for blood and marrow transplantation autoimmune diseases working party and collaborating partners. Bone Marrow Transplant.

[10] Fernández-Codina A, Walker KM, Pope JE (2018). Treatment algorithms for systemic sclerosis according to experts. Arthritis Rheumatol.

[11] Fischer A, Brown KK, Du Bois RM (2013). Mycophenolate mofetil improves lung function in connective tissue disease-associated interstitial lung disease. J Rheumatol.

[12] Frantz C, Huscher D, Avouac J (2020). Outcomes of limited cutaneous systemic sclerosis patients: results on more than 12,000 patients from the EUSTAR database. Autoimmun Rev.

[13] Fraticelli P, Fischetti C, Salaffi F (2018). Combination therapy with rituximab and mycophenolate mofetil in systemic sclerosis. A single-centre case series study. Clin Exp Rheumatol.

[14] Goh NS, Desai SR, Veeraraghavan S (2008). Interstitial lung disease in systemic sclerosis: a simple staging system. Am J Respir Crit Care Med.

[15] Highland KB, Distler O, Kuwana M (2021). Efficacy and safety of nintedanib in patients with systemic sclerosis-associated interstitial lung disease treated with mycophenolate: a subgroup analysis of the SENSCIS trial. Lancet Respir Med.

[16] Hoffmann-Vold AM, Fretheim H, Halse AK (2019). Tracking impact of interstitial lung disease in systemic sclerosis in a complete nationwide cohort. Am J Respir Crit Care Med.

[17] Hoffmann-Vold AM, Maher TM, Philpot EE (2020). The identification and management of interstitial lung disease in systemic sclerosis: evidence-based European consensus statements. Lancet Rheumatol.

[18] Hoyles RK, Ellis RW, Wellsbury J (2006). A multicenter, prospective, randomized, double-blind, placebo-controlled trial of corticosteroids and intravenous cyclophosphamide followed by oral azathioprine for the treatment of pulmonary fibrosis in scleroderma. Arthritis Rheum.

[19] Hughes M, Khanna D (2021). Rituximab for the treatment of systemic sclerosis: urgent need for an international randomised controlled trial. Lancet Rheumatol.

[20] Khanna D, Denton CP, Jahreis A (2016). Safety and efficacy of subcutaneous tocilizumab in adults with systemic sclerosis (faSScinate): a phase 2, randomised, controlled trial. Lancet.

[21] Khanna D, Lin CJF, Furst DE (2020). Tocilizumab in systemic sclerosis: a randomised, double-blind, placebo-controlled, phase 3 trial. Lancet Respir Med.

[22] Khanna D, Strek M, Southern B et al (2018) Expert consensus on the screening, treatment, and management of patients with systemic sclerosis-interstitial lung disease, and the potential role of anti-Fibrotics in a treatment paradigm for systemic sclerosis-interstitial lung disease: a Delphi consensus study. Arthritis Rheumatol 70

[23] Kowal-Bielecka O, Fransen J, Avouac J (2017). Update of EULAR recommendations for the treatment of systemic sclerosis. Ann Rheum Dis.

[24] Nadashkevich O, Davis P, Fritzler M (2006). A randomized unblinded trial of cyclophosphamide versus azathioprine in the treatment of systemic sclerosis. Baillieres Clin Rheumatol.

[25] Owen C, Ngian GS, Elford K (2016). Mycophenolate mofetil is an effective and safe option for the management of systemic sclerosis-associated interstitial lung disease: results from the Australian scleroderma cohort study. Clin Exp Rheumatol.

[26] Pakas I, Ioannidis JP, Malagari K (2002). Cyclophosphamide with low or high dose prednisolone for systemic sclerosis lung disease. J Rheumatol.

[27] Panopoulos ST, Bournia VK, Trakada G (2013). Mycophenolate versus cyclophosphamide for progressive interstitial lung disease associated with systemic sclerosis: a 2-year case control study. Am J Physiol.

[28] Park MC, Park YB, Jung SY (2004). Risk of ovarian failure and pregnancy outcome in patients with lupus nephritis treated with intravenous cyclophosphamide pulse therapy. Lupus.

[29] Parnham MJ, Nijkamp FP, Rossi A (2019). Nijkamp and Parnham’s principles of immunopharmacology.

[30] Shenoy PD, Bavaliya M, Sashidharan S (2016). Cyclophosphamide versus mycophenolate mofetil in scleroderma interstitial lung disease (SSc-ILD) as induction therapy: a single-centre, retrospective analysis. Arthritis Res Ther.

[31] Sircar G, Goswami RP, Sircar D (2018). Intravenous cyclophosphamide vs rituximab for the treatment of early diffuse scleroderma lung disease: open label, randomized, controlled trial. Rheumatology.

[32] Steen VD, Medsger TA (1998). Case-control study of corticosteroids and other drugs that either precipitate or protect from the development of scleroderma renal crisis. Arthritis Rheum.

[33] Suliman YA, Dobrota R, Huscher D (2015). Brief report: pulmonary function tests: high rate of false-negative results in the early detection and screening of scleroderma-related interstitial lung disease. Arthritis Rheumatol.

[34] Swigris JJ, Olson AL, Fischer A (2006). Mycophenolate mofetil is safe, well tolerated, and preserves lung function in patients with connective tissue disease-related interstitial lung disease. Chest.

[35] Tashkin DP, Elashoff R, Clements PJ (2006). Cyclophosphamide versus placebo in scleroderma lung disease. N Engl J Med.

[36] Tashkin DP, Roth MD, Clements PJ (2016). Mycophenolate mofetil versus oral cyclophosphamide in scleroderma-related interstitial lung disease (SLS II): a randomised controlled, double-blind, parallel group trial. Lancet Respir Med.

[37] Thiebaut M, Launay D, Rivière S (2018). Efficacy and safety of rituximab in systemic sclerosis: French retrospective study and literature review. Autoimmun Rev.

[38] Ueda T, Sakagami T, Kikuchi T (2018). Mycophenolate mofetil as a therapeutic agent for interstitial lung diseases in systemic sclerosis. Respir Investig.

[39] van Laar JM, Farge D, Sont JK (2014). Autologous hematopoietic stem cell transplantation vs intravenous pulse cyclophosphamide in diffuse cutaneous systemic sclerosis: a randomized clinical trial. JAMA.

[40] Volkmann ER, Tashkin DP, Li N (2017). Mycophenolate mofetil versus placebo for systemic sclerosis-related interstitial lung disease: an analysis of scleroderma lung studies I and II. Arthritis Rheumatol.

[41] Wu W, Jordan S, Graf N (2019). Progressive skin fibrosis is associated with a decline in lung function and worse survival in patients with diffuse cutaneous systemic sclerosis in the European scleroderma trials and research (EUSTAR) cohort. Ann Rheum Dis.

